# Obesity, dietary interventions and microbiome alterations in the development and progression of prostate cancer

**DOI:** 10.3389/fimmu.2024.1448116

**Published:** 2025-01-07

**Authors:** Shaun Trecarten, Michael A. Liss, Jill Hamilton-Reeves, John DiGiovanni

**Affiliations:** ^1^ Department of Urology, The University of Texas Health Sciences Center San Antonio, San Antonio, TX, United States; ^2^ Department of Urology, University of San Diego, San Diego, CA, United States; ^3^ Department of Urology, University of Kansas Medical Center, Kansas City, KS, United States; ^4^ Division of Pharmacology and Toxicology, College of Pharmacy, The University of Texas at Austin and Center for Molecular Carcinogenesis and Toxicology, The University of Texas at Austin, Austin, TX, United States

**Keywords:** cross-talk, microbiome, prostate cancer, obesity, diet obesity, diet, and role of microbiome in prostate cancer

## Abstract

**Purpose of review:**

The role of the microbiome in prostate cancer is an emerging subject of research interest. Certain lifestyle factors, such as obesity and diet, can also impact the microbiome, which has been implicated in many diseases, such as heart disease and diabetes. However, this link has yet to be explored in detail in the context of prostate cancer. The purpose of this review is to explore the cross-talk between obesity, dietary interventions, and microbiome alterations in the development and progression of prostate cancer.

**Recent findings:**

Many possible mechanisms exist linking obesity and dietary interventions to microbiome alterations and prostate cancer. The gut microbiome produces metabolites that could play a role in prostate cancer oncogenesis, including short-chain fatty acids, cholesterol derivatives, and folic acid. The microbiome also plays a pivotal role in the prostate tumor microenvironment (TME), contributing to inflammation, local tissue hypoxia, and epithelial-mesenchymal transition. A bidirectional relationship exists between obesity and the microbiome, and certain diets can enact changes to the microbiome, its associated metabolites, and prostate cancer outcomes.

**Summary:**

Cross-talk exists between obesity, dietary interventions, and the role of the microbiome in the development and progression of prostate cancer. To further our understanding, future human studies in prostate cancer should investigate microbiome changes and incorporate an assessment of microbiome-derived metabolites and cellular/immune changes in the TME.

## 1 Introduction

The human microbiome is a vast community of trillions of microorganisms inhabiting our epithelial surfaces, including skin, oral cavity, genitourinary tract, and gut (1). Each location has a unique composition of bacteria, viruses, fungi, and protozoa, and a careful balance is often achieved between the host and the microbiome (2). Dysbiosis occurs when there is a pathological shift in the usual harmony between microbiota and host, with alterations in taxonomic composition and local metabolites (1). Dysbiosis can follow from external factors, including obesity and diet, and has been implicated in diseases including heart disease, diabetes, rheumatoid arthritis, inflammatory bowel disease, and even cancer (3).

The microbiome’s contribution to the development and progression of prostate cancer is a recently growing area of research interest. While prostate tissue and urine microbiomes have been studied, special attention is paid to the role of the gut microbiome, which houses the largest population of commensal organisms, typically in the order of trillions (4). A gut-prostate axis has also been described and is hypothesized to play a bidirectional part in prostate neoplasia (4). With lifestyle factors such as obesity and dietary intake impacting the gut microbiome, further understanding of this relationship may help yield future interventions that improve prostate cancer outcomes (5, 6).

The purpose of this review is to highlight the complex interplay between obesity, dietary interventions and the role of the microbiome in the development and progression of prostate cancer. To link the concepts in a stepwise approach, this paper will discuss 1) the role of the gut microbiome in producing metabolites that may contribute to prostate cancer, 2) the interactions of the microbiome with the prostate tumor microenvironment (TME), and 3) the impact of obesity and individual diets on microbiome and prostate cancer.

## 2 Gut microbiome-derived metabolites and prostate cancer

Numerous metabolites related to the gut microbiome have demonstrated a role in prostate cancer, including short-chain fatty acids (SCFA), sex steroid hormones, bile acids, folic acid, trimethylamine, and phenylacetylglutamine (2). While viruses [e.g., human papilloma virus (7)] and protozoa [e.g., Trichomonas vaginalis (8–11)] may be associated with the development of prostate cancer, this section of the review will focus on those organisms that can produce metabolites assoctiated with prostate cancer, which will be connected later in this review with lifestyle factors and dietary interventions (see Section 4).

### 2.1 Short-chain fatty acids

SCFAs, examples of which include butyrate, propionate, and acetate, are generated from the fermentation process of dietary fiber by gut bacteria (12). Two major phyla are largely responsible for the development of SCFAs: butyrate is often produced by *Firmicutes*, and propionate and acetate are produced by *Bacteriodetes* (13). Acetate is largely the most abundant SCFA, but the ratio of acetate: propionate: butyrate varies in the literature (14). SCFAs, acting through G-protein coupled receptors, have ranging physiological functions with roles in inflammatory regulation (via anti-inflammatory interleukin-10 [IL-10] and pro-inflammatory IL-6), fat/energy metabolism, and cell-cycle regulation (13). For effects on the cell cycle, butyrate is the most studied SCFA, with anti-neoplastic mechanisms via p53 and p21 pathways, cyclin-dependent kinase-2 inhibition, and activation of other cell components, which can trigger apoptosis (15). After entry into colonocytes from the intestinal lumen, butyrate inhibits histone deacetylases (HDACs), resulting in histone hyperacetylation and induction of cell-cycle arrest and apoptosis (15). In prostate cancer, high expression of HDAC 1, 2, and 3 has been demonstrated (16). Furthermore, sodium butyrate has been demonstrated *in vitro* to lower androgen receptor gene expression in prostate cancer cells (17). However, the relationship of butyrate to cancer development is likely more complex, as high doses of butyrate were required for neoplastic inhibition in colorectal cancer. In contrast, cancer growth was promoted if concentrations were <5mM (18). *In vivo*, only a small proportion of gut bacteria-derived butyrate reaches the prostate since the liver consumes much of the SCFAs via the portal venous system (19).

While butyrate has been shown to induce growth inhibition and apoptosis in human prostate cancer cells *in vivo* (20), SCFA-producing bacteria (including *Rikenellaceae*, *Alistipes* and *Lachnospira*) were found to be more abundant in patients with high-grade disease, suggesting a role for SCFAs in the progression of prostate cancer (21). Another study by the same group demonstrated that SCFA supplementation (in the form of acetate, butyrate, and propionate) to phosphatase and tensin homologue (*Pten*) knockout mice enhanced tumor growth via insulin-like growth factor 1 (IGF1) potentiation through mitogen-activated protein kinase (MAPK) and phosphoinositide 3-kinase (PI3K) signaling (22). Furthermore, antibiotic mixture administration in mice led to reduced SCFA levels in feces, downregulation of both IGF1 and downstream MAPK and PI3K pathways, and inhibition of cancer growth (22). The authors proposed the possible existence of a gut-IGF1-prostate axis. The role of IGF1 in prostate cancer was also examined in a retrospective study comparing a cohort of men with acromegaly (N=2495) with a reference cohort (N= 4.3 million), revealing an increased risk of prostate cancer diagnosis (HR 1.33, [95%CI 1.09–1.63], p = 0.005) (23). A recent study examined the role of gut-derived SCFAs (acetate/butyrate mixture) in castrate-resistant prostate cancer, where there was evidence of enhanced invasion through autophagy by toll-like receptor 3 and chemokine CCL20, two mechanisms through which malignant cells can potentially evade host immune functions (24).

The relationship of SCFAs to prostate cancer is intriguing. SCFAs demonstrate pro- and anti-inflammatory abilities and can potentiate/inhibit tumor growth depending on concentration. Preclinical studies have shown conflicting results. Further research is required to expand our understanding of the impact of different SCFA mixtures/compositions, the role of prostatic SCFA concentrations, and the interplay with IGF1/IGF1R signaling.

### 2.2 Cholesterol derivatives

Cholesterol derivatives, such as bile acids (BAs) and sex steroid hormones, share a similar steroid nucleus and are reabsorbed via enterohepatic recycling (25). The gut microbiome can produce hormones from steroid metabolites, a concept coined the ‘sterolbiome’. Furthermore, the gut microbiome plays a significant role in regulating the excretion of steroids and their potency (26). It has been proposed that this interaction may also contribute to differences in pathology between biological sexes (27).

#### 2.2.1 Biles acids

Primary BAs are formed in the liver from cholesterol and, in humans, consist of cholic acid (CA), chenodeoxycholic acid (CDCA) as well as glycine- and taurine-bound derivatives (28). Bile salts, however, refer specifically to glycine/taurine bound BAs, which are present *in vivo* as anions (28). Primary BAs are metabolized into secondary BAs by the gut microbiome to the extent that the most prevalent BAs in feces are secondary BAs in the form of deoxycholic acid (DCA) and lithocholic acid (LCA) (28). Ursodeoxycholic acid (UDCA) is also a secondary BA.

There is a bidirectional relationship between BAs and the gut microbiome. Primary BAs function as strong surfactants, though, through dehydroxylation or deconjugation of glycine/taurine into secondary BAs, they become apolar and lose their toxicity to bacteria (29). Deconjugation occurs through bile salt hydrolases, and glycine/taurine can often be used as an energy source for bacteria (29). Bile salt hydrolases have been identified in many bacteria, including *Clostridium*, *Enterococcus*, *Bifidobacterium*, *Lactobacillus*, *Listeria*, and *Bacteroides* (29). For dehydroxylation, *Clostridiales*, *Eubacteria*, *Bacteroides*, and *Escherichia* have been shown to play a major role (29). Conversely, BAs can also regulate the gut microbiome’s composition and aid in bacterial translocation into tissues (29). Among other functions, bacteria can oxidize or epimerize BAs, and bacterial enzymes can produce secondary BAs (29). This complex interplay between BAs and the gut microbiome can create a secondary BA pool, which can then contribute to carcinogenesis/tumor suppression via downstream BA signaling (via cell membrane receptors such as G-protein coupled receptor and nuclear receptors such as farsenoid X receptor [FXR]) (29).

The role of bile acid in carcinogenesis is variable amongst different cancer sites, and for prostate cancer, CDCA, LCA and UDCA demonstrate tumor suppressor effects (29). CDCA has been shown to inhibit prostate cancer cell proliferation through potentiation of FXR (which inhibits the initial step in primary BA synthesis) and *Pten* (30, 31). LCA has been shown to promote prostate cancer cell apoptosis, autophagy, and mitochondrial dysfunction while UDCA demonstrates death receptor-mediated apoptosis (32, 33).

#### 2.2.2 Testosterone

Testosterone plays a significant role in the development of many diseases, including metabolic syndrome and prostate cancer. Testosterone is mainly produced in the Leydig cells of the testis and adrenal glands of males but demonstrates a close relationship to the gut microbiome. One study, which performed 16S rRNA sequencing on 54 patients with a negative prostate biopsy demonstrated a positive correlation (R_s_= 0.33, p=0.014) between relative *Firmicutes* abundance (irrespective of age, body mass index, or serum triglyceride or cholesterol) and serum testosterone level (34). Another study also utilized 16S rRNA sequencing to demonstrate that men with the highest tertile of serum testosterone (>455 ng/dL) had higher abundances of *Ruminococcus*, *Acinobacter*, *Dorea* and *Megammonas*, which also correlated positively with testosterone level (35). *Rumminococcus* had the strongest correlation (r=0.46, p=0.009) (35). Gut bacteria has also been implicated in the production of testosterone, with one study demonstrating that in a population of patients with castrate-resistant prostate cancer (CRPC), *Ruminococcus* can convert pregnenolone and hydroxypregnenolone into downstream androgens (36). Patients with CRPC had increased abundance of *Ruminococcus*, and if present, was associated with a worse prognosis (36). *Prevotella*, however, was associated with an improved prognosis (36). A proposed mechanism of the influence of *Ruminococcus* in unfavourable prostate cancer outcomes is via upregulation of lysophosphatidylcholine acyltransferase 1 (LPCAT1), which may contribute to neoplasia via DNA repair pathways, phosphatidylcholine remodeling, or mRNA synthesis and production of platelet activating factor (37). A further potential source of androgen is from organisms which express *desA* and *desB* genes (including *Clostridium scindens* and *Propionimicrobium lymphophilu)*, which enables conversion of cortisol to 11β-hydroxyandrostenedione via steroid-17,20-desmolase (38).

Overall, testosterone is associated with certain bacterial abundances, particularly *Ruminococcus*, which can play a role in the production of androgens and castrate-resistant prostate cancer.

#### 2.2.3 Estrogen

Estrogens are a family of sex hormones derived from cholesterol, and can be produced endogenously (estrone, estradiol, estriol), synthetically or from phytoestrogens (plant-based dietary sources of estrogen) (39). The concept of a functional estrobolome was presented based on the presence of bacteria that have the ability to metabolize estrogens, through action of β-glucuronidases involved in deconjugation (40). This increases circulating free estrogens, potentially affecting downstream proliferative mechanisms. After diffusion into the cell, gene expression is generally driven from the activated type of nuclear estrogen receptor (ER), with ER-α demonstrating a proliferative effect while ER-β exhibits an inhibitory effect on cell tissue (39). Indeed, ER-α activation leads to prostate neoplasia and osteoblastic tumorigenesis *in vivo*, and ER- β is less expressed in malignant cells compared to benign tissue (39, 41). However, the current literature regarding the impact of estrogens in prostate cancer cell proliferation has been criticized, since the most common human prostate cancer *in vitro* model (LNCaP cells) have a mutated androgen receptor which is activated by estradiol and has low ER expression overall (42).

In women, β-glucuronidase is present in bacteria such as *Bacterioides* and *Facealibacterium* and absent in *Eubacterium* (43). Secondary studies using shotgun sequencing compared male patients with and without prostate cancer, demonstrating increased relative abundance of *Bacteroides* in prostate cancer patients, whereas *Eubacterium* was relatively abundant in those with benign tissue (44). However, there was also an increased abundance of *Fecalibacterium prausnitzii* in the benign group, which may be attributed to its ability to produce butyrate from acetate (44). With growing knowledge of the sexual dimorphism between male and female gut microbiomes, further studies are required to examine the role of estrogen in the male gut microbiome. Preclinical models must also be optimized.

### 2.3 Folic acid

Folate in the diet is reduced with polyglutamate side chains, whereas folic acid exists in oxidized form as pteroylmonoglutamate (45). Absorption of dietary folate in the small intestine requires hydrolyzation of polyglutamated to monoglutamated folate, while folic acid as a supplement (in the monoglutamated form) is readily absorbed (46). Folic acid is reduced by hepatic dihydrofolate reductase (DHFR) and through the folate metabolic cycle as 5-methyltetrahydrofolate, aids with the conversion of homocysteine to methionine (46). This is subsequently converted to S-adenosylmethionine, which, as a primary methyl donor in many reactions, may potentiate genetic stability as its function in preventing neural tube defects but may also cause unregulated cell growth, resulting in neoplasia (46).

There are conflicting results in the literature regarding the role of dietary folate and folic acid in prostate cancer, though this is likely attributable to differences in folic acid fortification of cereal grains. A meta-analysis of 10 studies, including 202,000 men, revealed that high dietary folate had no significant impact on prostate cancer risk. In contrast, high serum folate levels were associated with an increased risk of prostate cancer (relative risk [RR]= 1.21, 95%CI-1.05-1.39 p=0.008) (47). For countries without folic acid fortification, one study from Sweden demonstrated that 90% of subjects had serum folate levels <11.1nM (48), while a Finnish study showed that 75% of cases had serum folate levels <10.8nM (49). Conversely, only 2.5% of U.S. men have serum folate levels less than 10.4nM (50). One case-control study (N= 6875 cases, 8104 controls) reported an increased odds of developing prostate cancer for the highest quintile of serum folate compared to the lowest quintile (odds ratio [OR] 1.13, 95%CI 1.02-1.26, p=0.018) (51). Serum folate level was also demonstrated to be associated with cellular proliferation in prostate cancer tissue, as measured by Ki67 staining (52). This study demonstrated that the mean Ki67 staining index in cancer tissue from patients with the highest quintile for serum folate (n=10; 117 ± 15nM) was 6.17 ± 3.2% versus 0.86 ± 0.92% for those in the lowest quintile (n=10; 18 ± 9nM, p< 0.0001) (52).

The microbiota within the gut are a potential source of folic acid. There is evidence that colonic bacteria produce significant amounts of folic acid which can participate in host metabolism (53). Furthermore, certain probiotics with strains of *Lactobacillus* and *Bifidobacterium* have been shown to produce folate (54). However, a separate metabolomic analysis demonstrated in patients with prostate cancer there was a reduced ability of the gut microbiome to produce folate (55).

### 2.4 Other metabolites

Other metabolites derived from gut microbiota include trimethylamine and amino acid metabolites, including hippuric acid, p-cresol sulfate, and phenylacetylglutamine (PAGIn).

Trimethylamine is metabolized from gut microbiota from betaine compounds (γ-butyrobetaine and crotonobetaine), choline, and carnitine and then is oxidized in the liver to form trimethylamine N-oxide (TMAO) (56). Choline, a precursor to trimethylamine, is derived from animal products and has been associated with prostate cancer lethality (57). Elevated levels of TMAO were linked to aggressive (i.e. TNM stage III-IV, Gleason score ≥8, AJCC ≥3) prostate cancer (OR 1.36) in a metabolomic analysis of the Alpha‐Tocopherol, Beta‐Carotene cancer prevention (ATBC) study, potentially through inflammatory mechanisms (58). However, metabolomic analysis of lethal (N=173) and non-lethal prostate cancer cases (N=519) from the Prostate, Lung, Colorectal, and Ovarian (PLCO) associated higher levels of choline and betaine with prostate cancer lethality, with higher TMAO levels having no significant impact (56). While choline may contribute to oncogenesis through providing an extensive supply of an essential component of cell membranes among other reasons, and betaine can provide a methyl group for S-adenosylmethionine, further evidence linking these metabolites to malignancy is conflicting (59).

The metabolomic analysis of the PLCO trial also associated PAGIn with prostate cancer lethality. PAGIn is a gut microbiome-derived product of the amino acid phenylalanine, and acts via adrenergic receptors, where it has been implicated in cardiovascular disease (60). Interestingly, β2 adrenergic receptor signaling has been associated with dysregulated apoptosis and increased prostate cancer cell invasion (61), though a recent meta-analysis found no impact of beta-blockers on prostate cancer mortality (62).

## 3 Microbiota and the prostate tumor microenvironment

While direct genotoxicity by colibactin-producing bacteria has been implicated as a mechanism for prostate carcinogenesis (63), recent work has been done to uncover the role of the prostate tumor microenvironment (TME), functioning as a symphony of many cell types, including supportive stromal cells, epithelial and endothelial cells, cancer-associated fibroblasts (CAF), and immune cells, including neutrophils, macrophages, and myeloid derived suppressor cells (MDSCs). Adaptive immunity (T and B lymphocytes) has also been described as a component of the prostate TME, though further discussion will be deferred to other sources as its role in obesity and dietary interventions is less well studied (64–68). There is an increasing understanding of the role of prostate microbiota within the TME and the numerous potential mechanisms and signaling pathways that may lead to neoplasia.

### 3.1 Inflammation

The complex interplay between microbiome-derived inflammation and the development and progression of many cancer types is challenging to dissect (69). For prostate cancer in particular, previous studies linking inflammation to neoplasia were potentially subject to detection bias, as prostatitis is associated with an elevated prostate-specific antigen (PSA) level and may therefore be more likely to be screened for prostate cancer (70). However, one study followed patients from the Prostate Cancer Prevention Trial (PCPT) who had a negative end-of-study prostate biopsy, who were then included in the Selenium and Vitamin E Cancer Prevention Trial (SELECT), and found that with increasing mean percentage of inflamed tissue, there was an increasing odds of developing prostate cancer (0–<1.8%, OR = 1.7; 1.8–<5% OR = 2.39; ≥5% OR = 3.31, p= 0.047) (71).

Certain organisms have been implicated in developing inflammation within the prostate or prostatitis. In a study of 16S rRNA sequencing comparing patients with benign and malignant prostate tissue, proinflammatory organisms from the urinary microbiome including *Streptococcus* and *Anaerococcus* were enriched in patients with prostate cancer (72). Organisms in the gut microbiome have also been linked to the development of prostatitis. A recent Mendelian randomization study using single nucleotide polymorphisms (SNPs) highly linked to 196 microbial taxa (N=1859 prostatitis, 72 799 controls) reported a causal role of four bacteria (*Fecalibacterium, LachnospiraceaeUCG004, Sutterella, and Gastranaerophilales)* in increasing prostatitis risk (73). Microbes in the prostate TME, through inflammatory interleukin (IL)-6, IL-8 and tumor necrosis factor alpha (TNF-α), can potentiate vascular endothelial growth factor (VEGF), which induces tumor cell proliferating pathways including nuclear factor kappa B (NF-κB) transcription factors, epidermal growth factor receptor (EGFR) and toll-like receptor (TLR) pathways (74).

### 3.2 Hypoxia

Due to the acidic metabolites and reactive oxygen species associated with early changes of prostate cancer, there is an element of tissue hypoxia (75). Hypoxia can result in genomic instability and loss of *Pten* (76). Additionally, hypoxia can impede the degradation of hypoxia-inducible factor 1 alpha (HIF-1α), driving angiogenesis and proliferation, leading to invasion, progression, and even resistance to radiotherapy and systemic therapy (76). Other mechanisms linked to malignancy include the PI3K/Akt/mTOR, NOX, Wnt/β-Catenin, and Hedgehog signaling pathways, meticulously outlined in a review by Mohammed et al. (75).

In contrast, the role of the microbiome in tissue hypoxia has also been elucidated as a potential treatment. By harnessing the ability of facultative anaerobes in hypoxic conditions, both *Salmonella typhimurium* and *Serratia marcescens* have been shown to trigger apoptosis in prostate cancer cells *in vitro* (77, 78). Oncolytic viruses (mammalian orthoreovirus) can also be employed to selectively target prostate cancer cells through HIF-1α inhibition, inducing apoptosis and downregulation of Akt, AR and PSA (74).

### 3.3 Epithelial-mesenchymal transition

The epithelial-mesenchymal phenotypic transition (EMT) of solid tumors confers cancer cells the ability to invade and metastasize (79). Fundamental to this process is the contribution of a variety of components within the TME, including cancer-associated fibroblasts (CAFs), tissue-associated macrophages (TAMs), myeloid-derived suppressor cells (MDSCs), and transforming growth factor β (TGF-β).

CAFs encompass the majority of tumor stroma and exhibit many functions depending on their subtype, including expressing genes for collagen formation (myofibroblastic CAFs), cytokine/chemokine secretion (inflammatory CAFs including IL-6 and IL-11) and antigen-presentation (apCAFs) via major histocompatibility complex (MHC) class II (80). CAFs may originate from resident fibroblasts through TGF-β activation, from trans-differentiation of adipocytes, or mesenchymal stem cells via CXCL-16 (81). After replacing the stromal tissue of the affected prostate, CAFs can induce extracellular matrix-remodeling enzymes with vimentin replacing E-cadherin, a structure vital for cell-cell junctions (82). The presence of CAFs has been associated with higher Gleason scores and worse biochemical-free and recurrence-free survival in prostate cancer (83).

TAMs are another predominant cell component of the TME and are generally classified as M1-type (proinflammatory, function to eliminate pathogens/tumors) and M2-type (anti-inflammatory, secrete IL-10 and TGF-β) (80). Within the TME, M2-type TAMs are more abundant compared to M1-type TAMs (84). TAMs, M2-type in particular, have been associated with prostate cancer progression, metastases, and resistance to androgen therapy (80). CAFs can also contribute to the polarization of TAMs to the M2-type by actions of IL-6 and stromal-derived growth factor 1 (SDF-1/CXCL12) (85). TAMs also potentiate angiogenesis through high expression of VEGF, TGF-β, and basic fibroblast growth factor (bFGF). Furthermore, often in response to bacteria, TAMs secrete matrix metalloproteinases (MMP-2 and MMP-9), resulting in enzymatic destruction of the basement membrane and stromal remodeling, which can result in phenotypic changes associated with EMT (86). However, some bacteria can have tumor suppressive properties, as one murine study observed that after intravenous injection of extracellular vesicles derived from *Akkermansia muciniphila*, there were increased tumor-killing M1-type macrophages and reduced M2-type (87). There was also an increase in granzyme-B positive and interferon γ-positive CD8+ T cells (87). One study performed single-cell RNA sequencing and found that a selection of TAMs had dysregulated lipid metabolism and that an abundance of lipid-loaded TAMs (from a high-fat diet [HFD]) was associated with tumor progression in a murine model (88). Our group has shown that obesity is associated with both TAM function and infiltration of adipose stromal cells (ASCs) producing CXCL12 and other chemokines that lead to increased progression of prostate cancer (89–92). The implication is that microbiome-based interventional studies addressing obesity could be developed targeting these pathways due to the long lead time of cancer development. Interestingly, TAM polarization may have implications for prostate cancer treatments and response to chemotherapy for those already with a diagnosis of prostate cancer (93).

Myeloid-derived suppressor cells (MDSCs) are a further key member of the TME, highly expressing inhibitory checkpoint molecules (e.g. PD-L1) and impacting T-cell function through production of reactive oxygen species and arginase-1 (94, 95). One murine study identified MDSCs as the main infiltrating cell type resulting in immune evasion (96), and MDSCs have also been shown to activate downstream androgen receptor pathways (through IL-23 secretion), thus contributing to castrate resistance (97). High circulating levels of MDSCs have been identified in prostate cancer patients compared to healthy patients (98) and are also associated with increased mortality (hazard ratio [HR] = 2.2, 95%CI 1.5–3.2) in a systematic review (5 studies, N= 235 patients) (99).

TGF-β plays a central role in EMT through its ability to polarize TAMs, activate CAFs to potentiate NF-κB signaling, and produce HIF-1, thus driving EMT (86). TGF-β is also implicated in the upregulation of Snail and P13K/Akt pathways, which can confer resistance of cancer cells to anoikis, a form of apoptosis trigged when cells detach from the ECM (100). It should be noted that TGF-β can act as an inhibitor of neoplasia in normal tissue and early stage disease (101).

### 3.4 Lipopolysaccharides

Lipopolysaccharide (LPS) is a component of the outer membrane of gram negative bacteria, and can function as an endotoxin after bacterial cleavage (102). Within the prostate TME, LPS can trigger toll-like receptor 4 (TLR4), which further potentiates pro-inflammatory cytokines including IL-6, TNF-α and TGF-β (103). Initially, TLR4 activation initiates an appropriate innate immune response to pathogens. Long-term activation of TLR4 signaling, however, can initiate malignant transformation and proliferation (103). One murine study demonstrated systemic LPS administration activated NF-κB signaling in DU145 prostate cancer cells *in vivo* (104).

Further evidence exists linking gut dysbiosis (rather than the prostate tissue microbiome) and LPS to prostate cancer. HFD leads to increased risk of prostate cancer progression and local inflammation is considered a critical component of the mechanism (105, 106). Under normal conditions, the gut barrier prevents the passage of endotoxins into the systemic circulation from the gut lumen (107). However, gut microbiome dysbiosis (induced from HFD) in *Pten*-knockout mice resulted in elevated serum LPS, pointing towards leakage of LPS from the gut into the bloodstream (108). Furthermore, administration of a histamine antagonist reduced IL6/STAT3 signaling and suppressed tumor growth (108). Another murine experiment reported that in antibiotic induced gut-dysbiosis with resulting enriched *Proteobacteria*, there was increased gut permeability (based on elevated serum LPS and colonic morphology) and intratumoral LPS (109). There was also demonstration of activation of the NF-κB-IL6-STAT3 axis, as well as *in vitro* and *in vivo* docetaxel resistance in mice with gut dysbiosis (109). Thus, LPS-induced inflammation in the prostate likely plays an important role in helping drive prostate cancer progression in certain dietary settings (i.e., HFD) that lead to gut dysbiosis.

## 4 Impact of obesity and individual diets on microbiome and prostate cancer

### 4.1 Obesity and high-fat diet

**Figure 1:**
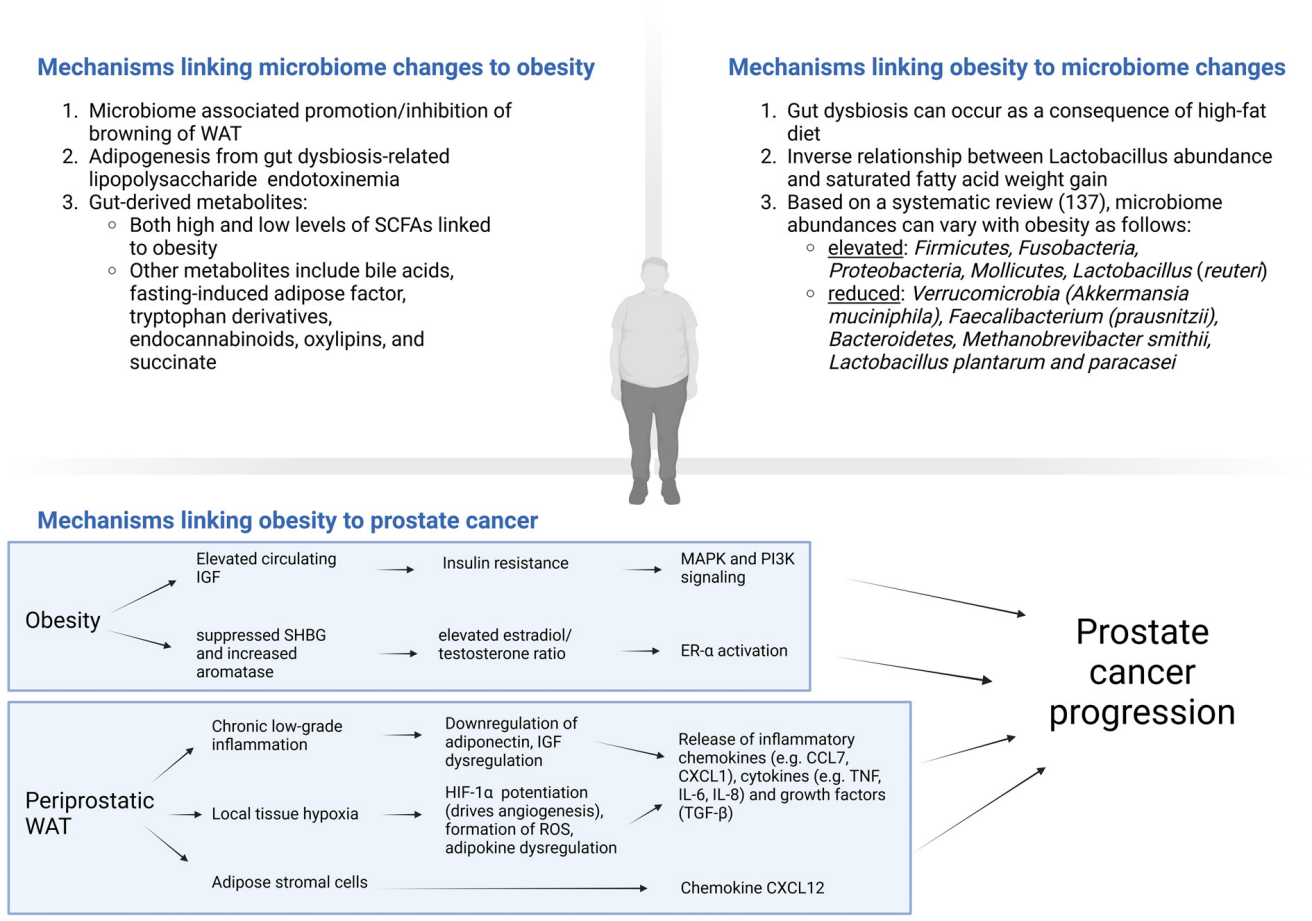
Cross-talk between obesity/high-fat diet and the microbiome, with mechanisms linking obesity to prostate cancer. CCL, CC chemokine ligands; CXCL, Chemokine (C-X-C motif) ligand (CXCL); ER, estrogen receptor; HIF, hypoxia-inducible factor; IGF, insulin-like growth factor; IL, interleukin; MAPK, mitogen-activated protein kinase; Pi3K, phosphoinositide 3-kinase; ROS, reactive oxygen species; SCFAs, short chain fatty acids; SHBG, sex-hormone binding globulin signaling; TGF, transforming growth factor; TNF, tumor necrosis factor; WAT, White adipose tissue. Created in BioRender. https://BioRender.com/y81u930.

Over the last few decades, the prevalence of obesity has more than doubled in most countries (110). While obesity is certainly related to many disease states, its connection to the development of *de novo* prostate cancer risk is conflicting (105). However, what is more clear is the relationship of obesity to prostate cancer progression, with increased primary treatment failure, progression, resistance to systemic therapy and mortality (105). Obesity often goes hand in hand with a HFD and has a bidirectional relationship with the microbiome (**Figure 1**). Furthermore, high carbohydrate diets and especially diets with fructose have been postulated to potentiate adiposity and prostate neoplasia (111–114). However, dietary interventions to suppress fructose in the context of prostate cancer have not yet been undertaken. Low carbohydrate diet is further discussed in Section 4.2.

#### 4.1.1 Mechanisms linking obesity to prostate cancer

Numerous potential mechanisms exist which link obesity to worse prostate cancer outcomes (115). Obesity can promote insulin resistance elevating circulating IGF levels, which as discussed previously, can promote carcinogenesis via MAPK and PI3K signaling pathways among others (115). Obesity is also thought of as a state of pathologic white adipose tissue (WAT) expansion and is associated with chronic low-grade inflammation, known to contribute to prostate cancer progression (116). After subcutaneous WAT stores are exhausted, excess adipose is stored as ectopic adipose tissue, examples of which include visceral adipose tissue or periprostatic adipose tissue (PPAT) (117). Ectopic fat in and of itself can increase insulin resistance, IGF axis dysregulation and downregulation of adiponectin, which results in an overall increase in inflammatory cytokine release (117). In patients with more PPAT, there is higher activity of MMP 2 and 9, which can enable progression (118). Furthermore, increased PPAT radiographically is associated with higher Gleason scores, advanced stage and shorter time to biochemical recurrence (119). Another mechanism in obesity includes hypoxia, which occurs from vascular stress from the excessive expansion of periprostatic white adipose. This leads to potentiation of HIF-1α which drives angiogenesis, and consequent reabsorption of dying adipocytes stimulates macrophage and leucocytes to secrete inflammatory chemokines (e.g. CCL7, CXCL1), cytokines (e.g. TNF, IL-6, IL-8) and growth factors (TGF-β) contributing to tumor growth and invasion (120–122). Infiltrating ASCs from perioprostatic WAT have also been shown to play a role in obesity driven systemic therapy resistance and prostate cancer progression via production of CXCL12 and other chemokines (90–92, 106). Obesity-induced WAT hypoxia can also result is dysregulation of adipokines, with evidence relating the consequent increased leptin and omentin-1 and decreased adiponectin with prostate cancer (115). This state of periprostatic WAT hypoxia in obesity may also result in the formation of reactive oxygen species and oxidative stress (123). Furthermore, antioxidants including nuclear factor erythroid 2-related factor 2 (Nrf2) and its target gene glutathione-S-transferase (GST) have been shown to be suppressed in prostate cancer, which may exacerbate the initial insult from obesity (124). Obesity is also related to circulating levels of sex steroids. While further study of the estrobolome is required to solidify its relationship to prostate cancer, previous studies have demonstrated a significant role of an elevated estradiol/testosterone ratio in prostate cancer pathogenesis (125). Obesity may contribute to this excess ratio by suppressing sex-hormone binding globulin and increased aromatase activity through excess adipose and hyperinsulinemia (115).

#### 4.1.2 Mechanisms linking microbiome changes to obesity

There is evidence that the gut microbiome can significantly impact the development of obesity, with certain microbial changes associated with the promotion or inhibition of browning of WAT (126). Some of the proposed mechanisms outlining the impact of the gut microbiome on obesity relate to the ability of the gut microbiome to produce certain metabolites that can affect energy metabolism.

SCFAs are one such example, which can provide approximately 10% of daily caloric intake from undigested fiber fermented by colonic bacteria (127). A complex relationship between SCFAs and obesity exists: elevated levels are absorbed in the liver and used as precursors for fatty acid/cholesterol synthesis (acetate) and gluconeogenesis (propionate) (128), whereas low SCFA levels have also been associated with obesity which can be corrected with SCFA supplementation (129). One proposed mechanism is through the regulation of satiety, as SCFAs stimulate the release of glucagon-like peptide 1 and peptide YY, both of which enhance satiety (i.e., low SCFAs result in increased energy intake) (128). Further complicating the relationship between obesity and SCFAs are the varying effects of individual SCFAs and differences in human adipocyte differentiation (via G-protein coupled receptor 43 [GPR43]) compared to murine models. Butyrate supplementation has demonstrated some benefits in one murine study, with associated fat oxidation and brown adipose activation with the prevention of the development of HFD-induced obesity and metabolic dysfunction through reduced food intake (130). However, the impact of SCFAs, and obesity is not entirely understood.

The integrity of the gut barrier can be disrupted by a number of factors, including excessive alcohol intake, HFD, or obesity itself (126). As discussed previously, metabolic endotoxemia can subsequently occur with leakage of LPS (among other gut-derived compounds) into circulation. The effect of LPS on adipogenesis can be variable. While some studies have linked adipocyte inflammation (via WNT–β-catenin–T cell factor 4 pathway) to hindered adipogenesis, others have linked LPS to increased preadipocyte proliferation and adipogenesis (via JAK–STAT and AMPK-dependent cPLA2 pathways) (126). However, previous studies have demonstrated that germ-free mice exhibit resistance to diet-induced obesity as well as reduced insulin resistance and WAT inflammation (126). Additionally, germ-free mice exposed to LPS bacteria demonstrated impaired glucose metabolism, increased macrophage accumulation, and increased M1-type macrophage (pro-inflammatory) polarization in the WAT (131). Other bacterial products, including peptidoglycans, lipopeptides, and flagellin, may also play a role and are discussed in a review by Cani et al. (126).

Bile acids (BAs) may also contribute to dysregulated glucose metabolism and obesity in various mechanisms (132). Equally, obesity can also increase BA synthesis, alter BA transport, and affect the overall BA composition (132). Furthermore, there may be a dual effect of secondary BAs on metabolic parameters, with one study demonstrating reduced insulin sensitivity with an antibiotic-associated depletion in *Firmicutes* abundance and secondary BA (133), and another study reporting associations of microbiome-associated increases in secondary BAs and non-alcoholic fatty liver disease (134). Through the action of BAs (especially CDCA) on Takeda GPR5 (TGR5), increases in brown adipose tissue activity and energy expenditure can also be seen, potentially counteracting obesity (135). Insulin sensitivity through increased GLP1 can also be induced by TGR5 (136). Other implicated metabolites include fasting-induced adipose factor (FIAF), tryptophan derivatives, endocannabinoids, oxylipins, and succinate (126).

#### 4.1.3 Mechanisms linking obesity to microbiome changes

Obesity can also impact the gut microbiome. Patients with obesity were shown in a systematic review of 32 studies to have an elevated *Firmicutes*/*Bacteroidetes* ratio, with higher abundance of *Firmicutes, Fusobacteria, Proteobacteria, Mollicutes, Lactobacillus* (*reuteri*), and less *Verrucomicrobia (Akkermansia muciniphila), Faecalibacterium (prausnitzii), Bacteroidetes, Methanobrevibacter smithii, Lactobacillus plantarum and paracasei* (137). One study reported that in obese *Pten*-knockout mice, HFD was associated with increased tumor growth and inflammatory cells associated with the TME including an increased M2:M1-type macrophage ratio, IL-6 and myeloid-derived suppressor cells (138). Inflammation and subsequent tumor growth was suppressed with anti-inflammatory medications (celocoxib) (138). The same group also administered antibiotics to *Pten*-knockout obese mice fed a HFD, and found significant alterations to the gut microbiome (namely *Rikenellaceae* and *Clostridiales*) with inhibition of prostate cancer proliferation (22). As discussed previously, the findings were hypothesized to occur due to reduced IGF1 from the antibiotic-associated reduction of SCFA producing gut bacteria (22). In human patients with obesity, there was increased tissue IGF1 (22). Compared to control-fed mice, HFD was found to cause gut dysbiosis as four measures of alpha-diversity were significantly reduced in the HFD group, with increased intestinal permeability (demonstrated by decreased zona occludens-1 production) and elevated serum LPS, possibly leading to prostate tumor growth through histamine signaling (108). Increased mast cell infiltration was also observed in prostatectomy specimens from obese patients (108). Another murine study compared an isocaloric HFD (enriched in saturated fatty acid) to a fish oil diet, and reported increased weight gain and prostate cancer progression with HFD (139). There was also an inverse relationship between *Lactobacillus* abundance and saturated fatty acid weight gain and prostate cancer progression (139).

### 4.2 Low carbohydrate diet

The role of a low carbohydrate diet (LCD) has been examined in prostate cancer. The rationale is based on its success as a weight loss diet (140), and also that in preclinical models, an LCD independent of weight loss is associated with reductions in prostate tumor growth and prolonged survival (141, 142). A *post-hoc* exploratory analysis of a study randomizing patients with biochemical recurrence after primary therapy for prostate cancer to either an LCD or diet as usual reported weight loss and improved PSA doubling time (PSADT) in those in the LCD group (30 vs. 13 months, p = 0.007) (143). LCD was also associated with reduced zonulin (an intestinal permeability marker), and there was a significant relationship between the degree of weight loss, and PSADT (144).

### 4.3 Mediterranean diet

The Mediterranean diet can exist in many variations on a common theme of 1) high intake of fish, high bioactive vegetables and fruits, nuts/seeds, and extra-virgin olive oil, 2) moderate intake of dairy and red wine, and 3) low intake of red meat, sugar, and processed products (145). There are numerous potential mechanisms through which it may reduce neoplasia, including 1) reduced inflammation from a reduction in saturated fatty acid intake and increased phytochemicals (see Section 4.5), 2) reduced oxidative stress, 3) increased insulin sensitivity from reduced caloric intake, 4) enhanced fecal mass estrogen excretion from high fiber (146). The Mediterranean diet has been extensively studied in many disease states, with associated gut microbiome changes reviewed recently in the context of obesity (147). The authors note significant microbiome variability amongst the reviewed literature examining the Mediterranean diet, but one common theme is the increase in butyrate and other SCFA producing bacteria (147). Interestingly, no SCFA changes were identified in studies that observed an increase in *Bifidobacterium* despite its known ability, highlighting the complexity of the cross-talk between diet and the gut microbiome (147). While some retrospective studies demonstrated improved cancer outcomes with a Mediterranean diet, a meta-analysis (10 studies, 33 451 patients) published in 2019 reported no relationship to prostate cancer risk or lethality (148). Thereafter, one retrospective study in 2021 demonstrated that higher adherence to a Mediterranean diet was associated with a lower risk of progression for men with prostate cancer on active surveillance (149). Conversely, an alternative Mediterranean diet was not associated with reductions in prostate cancer grade reclassification for patients in the Canary Prostate Active Surveillance cohort (150).

A measure of overall diet quality is the Health Eating Index (HEI), which reflects concordance of a particular diet with the Dietary Guidelines for Americans (151). Other dietary indexes exist, including the Alternate Healthy Eating Index (AHEI) and the Mediterranean Diet Scores (151). Using a plant-based dietary index, an analysis of the Healthy Professionals Follow-up Study (N=6655 men with prostate cancer) demonstrated that higher overall plant intake was associated with lower risk of prostate cancer lethality (152). A further case-control study demonstrated a reduced risk of prostate cancer in those adhering to a healthy eating pattern (153). Data from the National Institutes of Health–American Association of Retired Persons Diet and Health Study suggest that higher HEI-2005 and AHEI scores are associated with lower risk of prostate cancer in those who undergo PSA testing (154). In the previously mentioned Canary Prostate Active Surveillance cohort study, HEI-2015 was not associated with significant reductions in disease classification (150). The benefits of a healthy diet are clear, but its association with prostate cancer development and progression require more robust evidence before concrete conclusions can be made.

### 4.4 Omega-3 fatty acids

The Mediterranean diet is also rich in omega-3 fatty acids, which have been subject to significant research interest and may be protective in signaling pathways for inflammation, oxidative stress, and cell membrane composition. One study reported that supplementation of long-chain omega-3 fatty acid in mouse models with prostate cancer reduced tumor growth, gut *Ruminococcae* abundance, and fecal butyrate levels (155). However, the evidence for omega-3 fatty acids in prostate cancer is conflicting (156, 157). Furthermore, a recent phase IIb randomized trial comparing omega-3 supplements to placebo in prostate cancer patients pre-prostatectomy reported no difference in cancer proliferation (measured by Ki-67 expression) between groups (158).

### 4.5 Phytochemicals

Certain plant-derived natural products and phytochemicals have also been implicated in both the prostate TME and microbiome.

#### 4.5.1 Curcumin and urosolic acid

Curcumin, found in turmeric, is a hydrophobic polyphenol that has been shown to exert an anticancer effect through a reversal of p53 and *Pten*, attenuation of anti-apoptotic genes (e.g., OX-2, NF-κB, and Bcl-2) and regulation of the TME in favor of tumor suppression through inhibition of IL-10, TGF-β, CAFs, and TAMs (159). Another phytochemical, urosolic acid (UA), is a pentacyclic triterpenoid and is found in a variety of plant-based sources, including cranberries, apples, pears, lavender, basil, and rosemary (160). UA inhibits NF-κB and STAT3 activation in prostate cancer cells and suppresses prostate growth in xenograft murine models (161). A systematic review of the effects of curcumin and UA demonstrated that the most common affected pathways were NF-κB (n = 25 studies [14.5%]) and caspase 3/caspase 9 (n = 10 studies [41.6%]) for curcumin and UA respectively (162).

Both curcumin and UA can also regulate the abundance of gut bacteria. Curcumin was shown to elevate *Bifidobacterium* and *Lactobacillus* in an H22 liver tumor mouse model, reduce *Firmicutes/Bacteroides* ratio, and, when complexed to zinc, can reduce gut-dysbiosis related gut injury (163). In murine studies, UA was associated with beneficial gut bacteria and upregulated gut barrier tight junction proteins (ZO-1, occludin and claudin-1) (164). It has been postulated that curcumin and UA may have synergistic beneficial effects, and a phase 1 study recently examined the safety of combination curcumin (1200 mg/day) and UA (300 mg daily), demonstrating no grade 3 or 4 adverse effects (165). Furthermore, a combination of curcumin and UA was associated with a more favorable novel gut microbiome-derived risk score based on 10 aberrant metabolic pathways (165). Curcumin was also studied in one randomized trial where, compared to placebo in patients on intermittent androgen deprivation therapy, curcumin (1440 mg/day) suppressed PSA elevations and was well-tolerated (166). However, no significant improvements were seen in a phase II randomized study comparing curcumin (6 g/d for 7 days every 3 weeks) to placebo for men with metastatic castrate resistant prostate cancer on first-line docetaxel (167).

#### 4.5.2 Green tea

The major active ingredient in green tea is epigallocatechin-3-gallate (EGCG) (168). EGCG has been shown in preclinical studies to demonstrate anti-cancer activity through a variety of mechanisms, including decreases in IGF1, tumor-associated oxidative stress and angiogenesis, and androgen receptor antagonism (168–170). One study (N=26) reported that daily doses of Polyphenon E (containing 800 mg of EGCG among other tea polyphenols) in patients prior to prostatectomy had significant reductions in hepatocyte growth factor, vascular endothelial growth factor, and PSA (171). While a recent meta-analysis (7 studies, N=455) demonstrated no effect of green tea intake on PSA level (172), another meta-analysis demonstrated a significantly reduced pooled risk for prostate cancer development (risk-ratio = 0.41; 95% CI: 0.19- 0.86) in patients with high-grade prostate intraepithelial neoplasia (HGPIN) or atypical small acinar proliferation (ASAP) (173). One dose-response meta-analysis determined that higher green tea consumption (>7 cups/day) was linearly associated with a reduction in prostate cancer risk (174). The effect of green tea consumption on the gut microbiome has been studied, and has been associated with increased *Bifidobacterium* abundance (175, 176). Furthermore, in dextran sulfate sodium-induced colitis mouse models, oral EGCG enhanced gut barrier integrity and reduced inflammation with enriched *Akkermansia* and butyrate production (177). Furthermore, the gut microbiome can metabolize EGCG into phenolic acids and hydroxyphenyl valerolactones, which, along with EGCG, demonstrated anticancer activity in HCT-116 colon cancer cells (178).

#### 4.5.3 Soy products

Soybeans are a particularly rich source of isoflovanes, comprising of genistein, daidzein and glycitein in a 50:40:10 distribution and have implications in prostate cancer, respectively (179, 180). Genistein and daidzein are metabolized by gut microbiota glucosidases into secondary metabolites, though the exact metabolites can vary depending on the individual and abundance of particular gut organisms (181). For example, the secondary metabolites of daidzein include O-desmethylangolensin (O-DMA) and equol. However, It is estimated that daidzein can be converted to equol in only 20-35% of the Western population, compared to 60% of those in Asia (182). With a lower incidence of prostate cancer in Asian countries and evidence that prostate cancer risk can increase when those patients move to Western countries, it was thought that this phenomenon may be due to dietary differences (183).

These metabolites may exert antineoplastic activity through a variety of mechanisms. They can act as phytoestrogens, which are plant-based compounds with activity similar to estrogens and have an affinity for the ER- β receptor (182). Equol has also been shown to bind dihydrotestosterone, such that it cannot activate the androgen receptor (184). Other mechanisms include inhibition of IGF1, TGF-β, angiogenesis, MMPs involved in EMT, and Wnt/β-Catenin and NF-Kβ signaling pathways (182, 185). There is significant cross-talk between how isoflavones are metabolized, and how each metabolite can affect the gut microbiome diversity (186).

Despite a plethora of preclinical studies suggesting benefits for isoflavones in prostate cancer development and progression, there was little translation of similar results to human studies (187). A meta-analysis of 38 human studies demonstrated that there was no significant effect of soy/isoflavones on serum testosterone or estrogen levels (179). While another meta-analysis demonstrated that isoflavones also had no effect on PSA levels (188), one study thereafter demonstrated fermented soy was associated with significant PSA reductions in patients with elevated PSA and negative prostate biopsy (183). However, another study demonstrated that 6 month soy consumption (regardless of isoflavone content) was associated with reduced cancer incidence in men with HGPIN or ASAP (189). One meta-analysis analyzed risk of prostate cancer development with circulating isoflavone levels between Japanese and European men separately due to large differences between both cohorts (190). For European men, circulating levels were not associated with prostate cancer risk (190). For Japanese men with the lowest quartile of equol levels, there was a significantly lower risk for prostate cancer, though the overall trend of the relationship was not significant (190). There were no significant differences in rates of biochemical recurrence after radical prostatectomy in two studies randomizing soy protein consumption to placebo (187, 191).

### 4.6 Folic acid reduction diet

While the role of folic acid supplementation in prostate cancer is conflicting, one recent area of development is the effect of folic acid during androgen deprivation therapy. Prostate-specific membrane antigen (PSMA), also known as folate hydrolase 1, is a transmembrane protein that helps to facilitate folate absorption in the duodenum (192). During periods of androgen deprivation, PSMA is upregulated, and patients may be more susceptible to the cellular impact of folic acid during this period (46). One retrospective study found that during initiation of androgen deprivation therapy, 94% of men had increases in serum folate, compared to 67% during maintenance therapy (p=0.04) (193). Furthermore, a more rapid time to prostate cancer lethality was observed if serum folate increased to >200 ng/ml above baseline (P = 0.03) (193). This provided the rationale for a folic acid reduction diet, which was able to significantly reduce serum folate and red blood cell folate at 12 weeks (194). Further research is required to determine whether this may translate into improved prostate cancer outcomes. While preclinical models have shown some ability of folic acid supplementation to reduce obesity, there are conflicting results on its impact on the gut microbiome and cross-talk with SCFAs (195, 196).

## 5 Discussion

**Figure 2:**
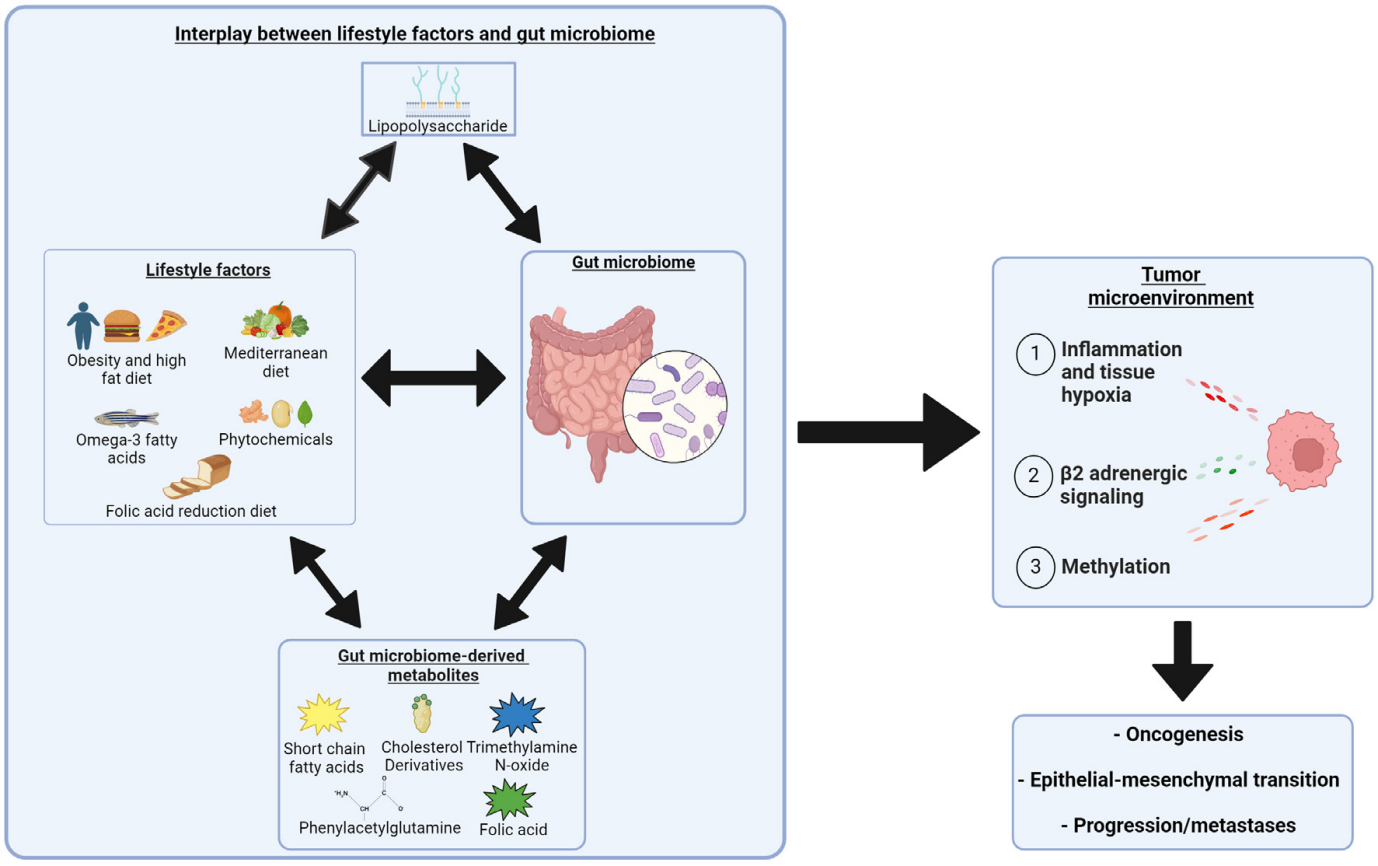
Complex interplay between obesity, dietary interventions, and the role of the microbiome in the development and progression of prostate cancer. Two headed arrows indicate a bidirectional relationship. Created with BioRender.com. https://BioRender.com/s65m081.

The role of the microbiome in prostate cancer is complex with significant cross-talk with metabolic changes associated with obesity and dietary interventions (**Figure 2**). Lifestyle factors and dietary interventions can alter microbiome diversity, and vary the production of gut microbiome-derived metabolites (SCFAs, cholesterol derivatives, folic acid, trimethylamine, and phenylacetylglutamine) that can contribute to prostate cancer growth. Finally, obesity and individual diets have been associated with microbiome diversity changes and produce metabolites and cellular response changes linked to prostate cancer development and alterations within the TME leading to progression.

While the role of the microbiome and its potential cross-talk with obesity and diet in prostate cancer is an exciting and growing area of research, significant challenges must be overcome before drawing strong conclusions. Some of the most compelling challenges are inherent to contemporary microbiome research, including heterogeneous methodologies that require ongoing appraisal and optimization to enhance standardization and reproducibility (197). As evidenced by the negative results of dietary interventions in humans (e.g., Mediterranean diet, HEI, omega-3 fatty acids, curcumin and UA and isoflavones) translation of preclinical non-human animal studies is also difficult. This may in part be due to differences in adipocyte differentiation and/or microbial abundances between murine models and humans. In the literature, it can be challenging in some studies to pinpoint the precise element of an intervention leading to change (e.g. confounding between obesity and HFD, SCFA mixtures with varied acetate:proprionate:butyrate ratios, individual phytochemical versus Mediterranean diet overall). Furthermore, a dual relationship can exist with some factors (e.g. obesity; TLR4 within the TME) initially offering protection to cancer development *de novo* but contributing to progression once malignancy is established. A further challenge is that few human studies in prostate cancer (likely limited by funding constraints) combine both microbiome analysis with an assessment of relevant microbial-produced metabolites and/or markers of interest systemically or within the TME. No easy solution exists, and it is only with continued and concerted effort to perform such analysis in human studies or ongoing trials that this complex interplay will become better understood.

## 6 Conclusion

In this review, we have explored the intricate cross-talk between obesity, dietary interventions, and the microbiome in the context of prostate cancer development and progression. Despite the promising insights gained, significant challenges remain in microbiome and metabolic research, particularly in standardizing methodologies and translating preclinical findings into clinical applications. Moving forward, a holistic and comprehensive approach to research is essential. This includes integrating multi-disciplinary strategies, leveraging advanced technologies, and fostering collaborations to unravel the complexities of these interactions. Such efforts will be crucial in developing effective preventive and therapeutic strategies for prostate cancer.
